# Effect of The Swimmer’s Head Position on Passive Drag

**DOI:** 10.1515/hukin-2015-0106

**Published:** 2015-12-30

**Authors:** Matteo Cortesi, Giorgio Gatta

**Affiliations:** 1 Department for Life Quality Studies, Rimini, School of Pharmacy, Biotechnology and Sport Science, University of Bologna, Bologna, Italy

**Keywords:** swimming, drag, hydrodynamic gliding, performance

## Abstract

The aim of this study was to investigate the effect of the head position on passive drag with a towing-line experiment in a swimming pool. The tests were performed on ten male swimmers with regional level swimming skills and at least 10 years of competitive swimming experience. They were towed underwater (at a depth of 60 cm) at three speeds (1.5, 1.7 and 1.9 m/s) and in two body positions (arms above the swimmer’s head and arms alongside the body). These two body positions were repeated while the swimmer’s head was positioned in three different ways: head-up, head-middle and head-down in relation to the body’s horizontal alignment. The results showed a reduction of 4–5.2% in the average passive drag at all speeds when the head was down or aligned to the swimmer’s arms alongside the body, in comparison to the head-up position. A major significant decrease of 10.4–10.9% (p < 0.05) was shown when the head was down or aligned at the swimmer’s arms above the swimmer’s head. The passive drag tended to decrease significantly by a mean of 17.6% (p < 0.001) for all speeds examined with the arms alongside the body position rather than with the arms above the head position. The swimmer’s head location may play an important role in reducing hydrodynamic resistance during passive underwater gliding.

## Introduction

In human swimming, the total drag is determined by the resistance forces acting opposite to the direction of travel, and the intensity of these forces is related to the speed. The term “passive drag” relates to the hydrodynamic resistance forces that occur when a swimmer remains in a stable position and is not moving any part of the body. The sums of all drag forces during swimming can be expressed by the passive hydrodynamic resistance in addition to the supplementary drag created by the movement of the swimmer’s body and limbs (Gatta et al., 2015). Several attempts have been made to quantify the total drag on swimmers. Passive drag measurements use the same theoretical approach (body in opposition to the water flow), while total drag studies employ systematically different methodologies (Havriluk, 2007). To date, this variability has led to controversial results in the scientific literature about the measurement of active drag; therefore, consistency is not a feature of active drag values (Zamparo et al., 2010). When the total drag is estimated based on data for passive drag and the frontal area is in opposition to the swimmer’s direction, the values are close to those of active drag, as measured by most authors (Zamparo et al., 2009).

Although the swimmer is in a stable prone position for a short time during a swimming competition, the majority of the starts and turns are performed in glide swimming, which is defined as a rigid streamlined body position used in passive towing (Guimaraes and Hay, 1985). The swimmer keeps a hydrodynamic position for as long as possible in the underwater phases to maintain speed. Guimaraes and Hay (1985) showed that the gliding period was the most important fraction of the swimming start and Vantorre et al. (2014) found that the efficiency of the glide phases was highly correlated with the time of the race and the starting performance. Moreover, as reported by Seifert et al. (2011), approximately 20% of the breaststroke is performed in glide swimming. Similar results by Chollet et al. (2006) pertain to the glide phase in the butterfly stroke. Therefore, a reduction of hydrodynamic resistance during the glide phase seems to be an important factor for swimmers performance.

Demonstrating the relevance of this phase, some authors have investigated the changes in body alignment and their correlation with gliding efficiency. Naemi and Sanders (2008) showed that glide efficiency was linked to the swimmer’s size and shape. However, to estimate the benefits of a more hydrodynamically stable position, it is necessary to understand the flow characteristics around the swimmer. Pendergast et al. (2006) noted that different flow velocities around the surfaces of the swimmer’s body could cause pressure changes. The changing zone of the flow velocity is called the ‘boundary layer’, and the flow separation and transition from laminar to turbulent depend on uniform velocity and, consequently, a constant pressure. Given that the pressure distribution over the body is the dominant factor of the swimmer’s passive drag (Marinho et al., 2009), keeping the boundary layer attached to the swimmer seems to be very important for reducing flow resistance (Polidori et al., 2006). Pendergast et al. (2006) suggested that separation of the boundary layer occurred near the curvatures and circumference of the swimmer’s body and that the change of surfaces at the head, back and buttocks could create adverse pressure gradients that would result in an increase of the swimmer’s drag. In addition, a previous study showed that a laminar flow in a swimmer’s glide profile was disrupted at the head (Mollendorf et al., 2004). It seems that the head position represents the first point of significant shape changes when the swimmer is in a streamline gliding position.

Some studies have examined changes in the head position during underwater gliding. Zaidi et al. (2008) evaluated the effect of three head positions (aligned with the body, lifted up or lowered) on hydrodynamic resistance using computational fluid dynamics (CFD) methodology. The authors showed that the head aligned with the axis of the body induced a decrease in the drag of approximately 20% at high swimming velocities. The same results were found by Popa et al. (2011; 2012) who confirmed that the position of the head aligned with the body provided less resistance in underwater glide swimming.

As proposed by several authors (Mollendorf et al., 2004; Pendergast et al., 2006; Popa et al., 2011; Zaidi et al., 2008), changes in the head position can affect the hydrodynamic performance of the glide. To our knowledge, only studies with CFD analysis have examined the quality of gliding related to the swimmer’s head position. As proposed by Marinho et al. (2009), some restrictions inherent in the use of two- or three-dimensional steady flow models and the assumption that the fluid around the swimmer is laminar or turbulent must be considered when analysing results with CFD. The purpose of our study was to investigate the influence of the head position on passive drag by a direct pool experiment with a swimmer’s in-line towing.

## Material and Methods

### Participants

A total of ten male swimmers (age: 21 ± 2 years; body height: 1.80 ± 0.06 m; body mass: 75.9 ± 6.9 kg) participated in this study after giving their informed consent. All of the swimmers were regional-level and had at least 10 years of competitive swimming experience. The investigation was performed during the winter of 2014, when the swimmers were in the competition period.

The study conformed to the standards set by the Declaration of Helsinki, and the procedures were approved by the Bioethics Committee of the University of Bologna. The swimmers were informed about the procedures, potential risks and benefits of the study.

### Passive drag measurements

The swimmers’ passive drag was measured using an electro-mechanical device (Swim-Spektro, Talamonti Spa, Ascoli Piceno, Italy). A low voltage isokinetic engine anchored static at the edge of the pool measured the force (N) needed for towing the swimmer. Each participant was connected to the machine via a non-elastic wire and was towed at a programmed speed.

The distance traversed by the swimmer in passive towing was 20 m in length at a constant depth of 60 cm below the water surface. The control of the depth was conducted by the passage within three rings of 90 cm diameter anchored to the swimming pool bottom. The rings were placed in the path of the towed swimmer at 10, 15 and 20 m after the starting point. A pulley support attached to the starting wall was used to ensure an in-line tow. For further analysis, we considered the data acquired between the first and third rings when the speed was constant. The data acquisition system was linked to a PC and controlled via dedicated software (DB:4, Talamonti Spa, Ascoli Piceno, Italy), and the device was calibrated before each experimental session.

### Experimental Procedures

The 10 swimmers were in the pool for the single test session for average duration of 2.5 hours. The trials were conducted in the morning in a 25 m indoor swimming pool with average water temperature of 28.0 ± 0.5º C. The study protocol was divided into three parts:

- Anthropometric measurements (body height and mass) were performed after a 15 -min swimming warm-up.
- Before the data acquisition, the swimmer performed two towing trials in each condition described in the “swimmer’s head and body position” section to become familiar with the test.
- The participant performed the main session of the test procedure. The passive towing protocol was conducted for one swimmer at a time. The order in which the three head position tests were performed was counterbalanced across swimmers, to exclude the effects of sequence noise, as follows: A-B-C (n = 4 swimmers), C-A-B (n = 3 swimmers), and B-C-A (n = 3 swimmers). Five swimmers started this test sequence from the first body position, and five swimmers started from the second body position. Technical suggestions during the trials were deliberately avoided in order to not affect the test participants.

### Swimmer’s head and body positions

The basic stable prone position was performed while the subject assumed the best hydrodynamic glide position during towing under the water surface. The lower limbs and feet were held at maximum extension. During passive towing, the swimmer was required to maintain the best hydrodynamic glide position defined by the protocol for the entire trial. Each test began following a maximal inspiration by the participant, who then held his breath during towing.

**Figure 1:**
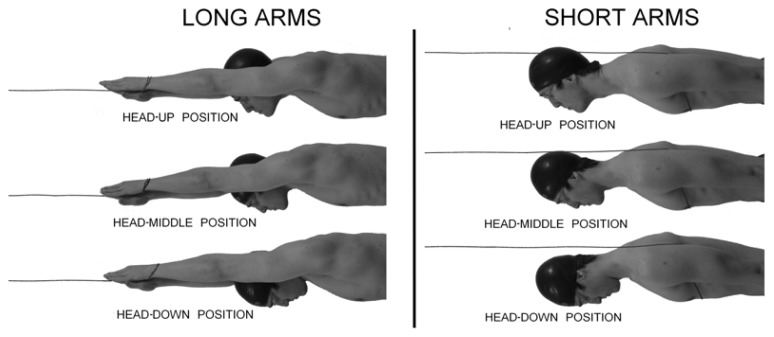
Schematisation of the three head positions and the two body positions used in the study

The glide prone position was repeated with the three different swimmer’s head positions described below (Figure 1):

- Head-up (HU): the swimmer looked directly forward, and his ears were above the forearms and fully extended over the head.
- Head-middle (HM): the swimmer looked downward at 90 degrees to the swimming direction, in a neutral position. The swimmer’s ears were covered by the forearms, which were fully extended over the head.
- Head-down (HD): the swimmer looked backwards, and his ears were below the forearms, which were fully extended over the head.

The participant performed each head position trial at the three different speeds that are most used in swimming competition (1.5, 1.7, 1.9 m/s). The three different speeds for each swimmer’s head position were repeated in two prone body positions. In the first position, the “long arms” (LA), the swimmer assumed the best hydrodynamic prone position, and the arms were extended over the head with one hand over the other. In the second body position, the “short arms” (SA), the same basic glide position was assumed, with the upper arms positioned along the body and the hands in contact with the sides of the thighs.

When the swimmer was in the LA position, he was towed from the device linked to the wrists. In this manner the cable was anchored around the wrist of the swimmer’s hand that was positioned above the other, without affecting the streamline position. In the SA position, the wire of the passive drag device turned around the swimmer’s trunk at the underarms level. Also for this modality, a connection mode to the swimmer that did not affect the hydrodynamic prone position of the swimmer was chosen (Figure 1).

The swimmer’s body position during the glide was carefully checked by one operator positioned outside the pool. To assess the test reliability, the passive towing trial was repeated five times for each swimmer’s head position, body position and speed, for a total of 90 trials per participant.

### Statistical analysis

To assess general patterns of the effects, the overall mean and standard deviation of each variable were calculated for all passive drag trials. For all of the variables (the swimmer, head and body positions, speed), the value of the coefficient of variation (CV) did not exceed 15.1%. This result indicates that the mean of the 5 trials was reliable and could be used for further analysis.

The data on passive drag were analysed in two ways. First, to evaluate differences among the swimmer’s head position, the body position, speed and their interaction, 3-way repeated measures ANOVA was used. Second, to analyse the differences between head positions that were found to be significant with ANOVA, simple pairwise multiple comparison procedures were performed with the Tukey post hoc test. The level of significance was set at *p* < 0.05, and Cohen’s *d* was reported for pairwise comparisons as a measure of effect size. The statistical analyses were performed with SPSS Statistics Rel. 14.0.0 (SPSS, Chicago, IL, USA).

## Results

Mean and standard deviation values of the passive drag (N) are described in Tables 1 and 2. The tables report individual data at the three considered speeds (1.5, 1.7 and 1.9 m/s) for the three head positions (HU, HM and HD) and the two body positions (SA and LA).

ANOVA revealed significant differences in the following variables: speed (F_(2, 16)_ = 683,932, *p* < 0.001), the body position (F_(1, 8)_ = 115,409, *p* < 0.001), the head position (F_(2, 16)_ = 7,116, *p* < 0.05), speed ×body position (F_(2, 16)_ = 29,448, *p* < 0.001) and speed ×head position (F_(4, 32)_ = 2,754, *p* < 0.05).

Passive drag was significantly larger for all of the speeds examined in the SA than in the LA position (*p* < 0.001). The mean reduction of passive drag was 17.6% in the LA condition. Moreover, the results showed a significant difference for passive drag (*p* < 0.001) at the three levels of speed (1.5 vs. 1.7, 1.5 vs. 1.9, 1.7 vs. 1.9 m/s).

**Figure 2:**
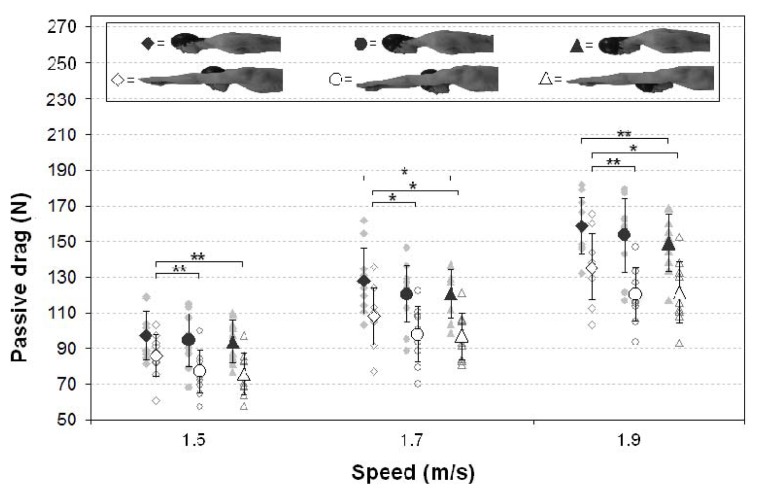
Overall mean (large symbols), individual data (small symbols) and standard deviation for passive drag. The passive drag of the swimmer’s head-up, head-middle and head-down positions is shown, respectively, with diamonds, circles and triangles. Open symbols represent the long arms position and the close symbols, the short arms position (^*^p < 0.05; ^**^p < 0.01)

Regarding the swimmer’s head position, passive drag was found to be significantly lower in the LA condition (with the arms in front of the head) for the HD and HM than the HU at all speeds (Figure 2). No differences were observed in passive drag between the HD and HM at all speeds. The statistical analysis revealed that in the SA condition (with the arms alongside the body), significant highest values of passive drag were only in the HU rather than in the HD at the two fastest speeds (1.7 and 1.9 m/s). However, the pairwise comparison showed no significant differences for the HM rather than for the HU and HD at all speeds. When the comparison was significant, the effect size of swimming was large (mean Cohen’s *d* = 0.72).

Therefore, the results show a reduction of 4–5.2% in average passive drag when the head was down or aligned with the swimmer’s arms alongside the body, in comparison to the head-up position. There was a major decrease of 10.4–10.9% in passive drag when the head was down or aligned with the swimmer’s arms above the swimmer’s head.

## Discussion

This study aimed to investigate the effect of the swimmer’s head position on passive drag during underwater towing. We considered three head positions, i.e. head-up, head-middle and head-down, with respect to the body’s horizontal alignment. Additionally, the passive body positions most used in glide swimming (with upper arms extended above the head or alongside the body) were also investigated. This is the first time that the swimmer’s head position has been investigated in a direct pool experiment during passive in-line towing in two stable body positions commonly used in glide swimming.

Recent studies (Cortesi et al., 2014; Gatta et al., 2013) have shown that athletic gear worn by swimmers, such as full-body suits and swim caps, may affect the swimmer’s body shape and passive drag. It has been previously shown that the shape of the swimmer, determined by the cross-sectional area, is a decisive factor affecting hydrodynamic resistance (Mollendorf et al., 2004; Zamparo et al., 1996). Indeed, passive drag resistance during underwater towing with the trailing arms along the body seems increased by 30–60% compared with the streamline glide position (Bulgakova et al., 2001; Marinho et al., 2009; 2011). However, in our study, increased hydrodynamic resistance due to the LA position was lower by approximately 20%. These different results could be due to the different methodologies used and theoretical assumptions required by different models (for example, for CFD, which was used in previous studies, the zero roughness relates to the swimmer’s human skin). Our results have practical applications for swimmers, such as the advantage for the breaststroke start and turn, where more time is spent in the first glide (before the arm pull) than in the second glide position.

The LA (streamlined) position seems to smooth the swimmer’s anatomical shape especially at the head (Marinho et al., 2011) and when the ears are pressed by the upper arms and shoulders (Zatsiorsky, 2000). In our study, head lifting during streamlined underwater gliding in respect to the middle or down position of the head caused a 10% average increase in passive drag. Previous studies using CFD analysis showed that the head-middle was the optimal position in the underwater glide when the arms were extended at the front (Popa et al., 2011; Zaidi et al., 2008). The authors showed a change of 20% of the swimmer’s hydrodynamic resistance when vertical shifts of the head from the position of horizontal alignment were carried out. Despite the validity and accuracy of the approaches used in swimming research (Bixler et al., 2007), the slight differences in passive drag values between previous studies and ours could be due to limitations of the commonly used CFD methodology (Marinho et al., 2009). Several authors have demonstrated that each slight displacement of the head position from horizontal alignment at flow velocities of 1.7–2.0 m/s causes increases in total hydrodynamic resistance from 2 to 40% (Miyashita and Tsunoda, 1978). In the present study, the high drag difference could be due to the methodology of passive drag measurement. Gliding was performed on the surface (Lyttle et al., 1998), and the use of the water tunnel could have produced a less satisfying comparison (Bixler et al., 2007).

As confirmed by Zaidi et al. (2008), a lower deficit reduction in the flow velocity around the head occurs when the head is aligned horizontally. In this position, the head is more covered by the arms than in any of the other positions analysed. This phenomenon explains the benefit in hydrodynamic resistance verified in our study, which shows that the aligned head seems to allow the swimmer to perform the best penetration in underwater gliding. In our study, the lower head position, as described in the literature, seems to be the least favourable position. When comparing Figure 1 with the figures shown in another study (Popa et al., 2011; Zaidi et al., 2008), it is clear that the standardisation of the head-down position defined in our protocol shows less displacement from the horizontal alignment. This phenomenon could produce a minor obstacle against the oncoming flow and could thus reduce hydrodynamic resistance.

Even correct alignment of the body segments during the glide position with the arms along the sides can affect the swimmer’s speed (Gatta et al., 2015; Vilas-Boas et al., 2010). To our knowledge, no author has investigated the relationship between the passive drag with arms alongside the trunk and the swimmer’s head position. The present study showed lower reductions in drag when keeping the head in a horizontal position compared to the same speed in gliding with the arms extended over the head. Mollendorf et al. (2004) showed that the laminar flow in a swimmer glide profile was disrupted at the head, most likely due to the head position during underwater streamline gliding. In our opinion, the lower effect of a different head position in gliding with arms along the sides could be explained by smoothing of the flow effect produced by the extended arms over the head. The swimmer’s shape influences the ratio of inertial to viscous (friction) forces and affects the point where the boundary-layer flow shifts from laminar to turbulent or separates from the body (Webb, 1975). Raising the arms over the head during gliding could provide, due to the displacement of the head from the aligned position, a major influence on the passive drag, thus changing the flow characteristics around the swimmer.

Despite high reliability of the test, flexibility of the human body structure does not ensure streamline alignment as a mannequin simulation. This is certainly the major limitation of this study. Future research with techniques of computational analysis or drag measurements with a towed mannequin should investigate the influence of the head position on passive drag.

In conclusion, this study was based on the assumption that the measurement of hydrodynamic resistance when a swimmer was towed and inactive would be a reliable methodology to investigate the swimmer’s drag (Havriluk, 2007). Our results suggest that performance improvements could be achieved from changes in the body’s position in the water. The swimmer’s head location seems to play an important role in reducing hydrodynamic resistance during gliding.

## Figures and Tables

**Table 1:** Individual data on passive drag for each head position (HU, HM and HD) and speed (1.5, 1.7 and 1.9 m/s) in the short arms (SA) position

	Passive Drag (N) in SA position								
	
Subject	1.5 m/s			1.7 m/s			1.9 m/s		
	HU	HM	HD	HU	HM	HD	HU	HM	HD
1	89.2	112.9	103.4	134.5	136.5	136.9	172.3	179.3	160.5
2	118.2	115.0	108.9	154.9	146.8	129.8	179.2	177.5	168.6
3	102.0	95.4	97.5	123.9	126.7	129.6	156.8	157.2	156.0
4	86.8	97.1	87.5	119.6	124.0	111.8	148.9	162.6	144.5
5	81.4	78.7	76.7	103.6	95.3	98.6	132.3	117.0	117.4
6	119.5	107.1	110.3	162.2	129.1	137.5	182.1	156.3	169.1
7	98.8	95.6	101.4	126.5	127.7	127.5	158.5	163.2	151.4
8	104.1	86.9	85.3	131.4	112.8	119.2	166.8	158.6	152.3
9	88.8	90.3	84.4	114.1	118.8	112.2	147.2	143.0	138.8
10	84.8	67.8	81.3	110.6	88.3	103.9	146.1	121.5	134.0
Mean	97.4	94.7	93.7	128.1	120.6	120.7	159.0	153.6	149.2
SD	13.6	14.8	12.1	18.6	17.8	13.6	16.0	20.9	16.1

**Table 2:** Individual data on passive drag for each head position (HU, HM and HD) and speed (1.5, 1.7 and 1.9 m/s) in the long arms (LA) position

	Passive Drag (N) in LA position								
	
Subject	1.5 m/s			1.7 m/s			1.9 m/s		
	HU	HM	HD	HU	HM	HD	HU	HM	HD
1	93.2	84.5	83.1	110.9	108.7	105.5	134.3	127.2	138.5
2	97.4	82.4	83.6	124.0	100.9	105.8	160.5	133.0	132.8
3	87.8	72.1	70.9	108.8	96.7	91.4	138.2	113.7	121.6
4	91.1	82.6	69.0	113.0	107.3	93.1	143.9	121.5	107.9
5	60.9	57.4	57.6	77.1	70.3	81.0	103.5	93.8	93.2
6	103.5	99.7	97.0	135.7	122.4	106.8	165.6	147.0	130.5
7	82.6	84.4	77.1	105.0	110.7	98.5	129.8	133.2	115.7
8	85.8	73.7	85.7	107.7	93.7	121.3	134.4	116.2	152.7
9	75.9	69.7	69.8	91.4	88.5	83.9	112.8	115.5	110.8
10	82.0	64.6	63.8	106.0	79.3	83.0	136.8	104.9	112.4
Mean	86.0	77.1	75.8	107.9	97.8	97.0	136.0	120.6	121.6
SD	11.9	12.1	11.8	16.0	15.6	12.9	18.8	15.3	17.3

## References

[b1-jhk-49-37] Bixler B, Pease D, Fairhurst F (2007). The accuracy of computational fluid dynamics analysis of the passive drag of a male swimmer. Sports Biomech.

[b2-jhk-49-37] Bulgakova NZh, Afanasev VZ, Makarenko LP, Morozov SN, Popov OI, Chebotareva IV (2001). Swimming.

[b3-jhk-49-37] Chollet D, Seifert L, Boulesteix L, Carter M (2006). Arm to leg coordination in elite butterfly swimmers. Int J Sports Med.

[b4-jhk-49-37] Cortesi M, Fantozzi S, Di Michele R, Zamparo P, Gatta G (2014). Passive drag reduction using full-body swimsuits: the role of body position. J Strength Cond Res.

[b5-jhk-49-37] Gatta G, Zamparo P, Cortesi M (2013). Effect of swim cap model on passive drag. J Strength Cond Res.

[b6-jhk-49-37] Gatta G, Cortesi M, Fantozzi S, Zamparo P (2015). Planimetric frontal area in the four swimming strokes: Implications for drag, energetics and speed. Hum Mov Sci.

[b7-jhk-49-37] Guimaraes A, Hay J (1985). A mechanical analysis of the grab starting technique in swimming. Int J Sport Biomech.

[b8-jhk-49-37] Havriluk R (2007). Variability in measurement of swimming forces: a meta-analysis of passive and active drag. Res Q Exerc Sport.

[b9-jhk-49-37] Lyttle AD, Blanksby BB, Elliot BC, Lloyd DG (1998). The effect of depth and velocity on drag during the streamlined glide. J Swim Res.

[b10-jhk-49-37] Marinho DA, Reis VM, Alves FB, Vilas-Boas JP, Machado L, Silva AJ, Rouboa AI (2009). Hydrodynamic drag during gliding in swimming. J Appl Biomech.

[b11-jhk-49-37] Marinho D, Barbosa T, Rouboa A, Silva A (2011). The hydrodynamic study of the swimming gliding : a two-dimensional computational fluid dynamics (CFD) analysis. J Hum Kin.

[b12-jhk-49-37] Miyashita M, Tsunoda R (1978). Swimming medicine IV.

[b13-jhk-49-37] Mollendorf JC, Termin AC, Oppenheim E, Pendergast DR (2004). Effect of swim suit design on passive drag. Med Sci Sports Exerc.

[b14-jhk-49-37] Naemi R, Sanders R (2008). A “hydrokinematic” method of measuring the glide efficiency of a human swimmer. J Biomech Eng.

[b15-jhk-49-37] Pendergast DR, Mollendorf JC, Cuviello R, Termin AC (2006). Application of theoretical principles to swimsuit drag reduction. Sports Eng.

[b16-jhk-49-37] Polidori G, Taiar R, Fohanno S, Mai TH, Lodini A (2006). Skin-friction drag analysis from the forced convention modeling in simplified underwater swimming. J Biomech.

[b17-jhk-49-37] Popa CV, Zaidi H, Arfaoui A, Polidori G, Taiar R, Fohanno S (2011). Analysis of wall shear stress around a competitive swimmer using 3D Navier-Stokes equations in CFD. Acta Bioeng Biomech.

[b18-jhk-49-37] Popa CV, Arfaoui A, Fohanno S, Taıar R, Polidori G (2012). Influence of a postural change of the swimmer’s head in hydrodynamic performances using 3D CFD. Comput Methods Biomech.

[b19-jhk-49-37] Seifert L, Chollet D, Mujika I (2011). World book of swimming: From Science to Performance.

[b20-jhk-49-37] Vantorre J, Chollet D, Seifert L (2014). Biomechanical analysis of the swim-start: a review. Sports Sci Med.

[b21-jhk-49-37] Vilas-Boas JP, Costa L, Fernandes R, Ribeiro J, Figueiredo P, Marinho D, Silva A, Rouboa A, Machado L (2010). Determination of the drag coefficient during the first and second glide positions of the breaststroke underwater stroke. J App Biomech.

[b22-jhk-49-37] Webb PW (1975). Hydrodynamics and energetics of fish propulsion.

[b23-jhk-49-37] Zaidi H, Taıar R, Fohann S, Polidori G (2008). Analysis of the effect of swimmer’s head position on swimming performance using computational fluid dynamics. J Biomech.

[b24-jhk-49-37] Zamparo P, Capelli C, Termin AB, Pendergast DR, di Prampero PE (1996). The effect of the underwater torque on the energy cost, drag and efficiency of front crawl swimming. Eur J Appl Physiol O.

[b25-jhk-49-37] Zamparo P, Gatta G, Capelli C, Pendergast DR (2009). Active and passive drag, the role of trunk incline. Eur J Appl Physiol.

[b26-jhk-49-37] Zamparo P, Capelli C, Pendergast DR (2010). Energetics of swimming: A hystorical perspective. Eur J Appl Physiol.

[b27-jhk-49-37] Zatsiorsky V (2000). Biomechanics in Sport.

